# Molecular Cloning and Characterisation of a Novel Type of Human Papillomavirus 160 Isolated from a Flat Wart of an Immunocompetent Patient

**DOI:** 10.1371/journal.pone.0079592

**Published:** 2013-11-08

**Authors:** Tsuyoshi Mitsuishi, Ikuroh Ohsawa, Toshihiko Kato, Nagayasu Egawa, Tohru Kiyono

**Affiliations:** 1 Department of Dermatology, Tokyo Women’s Medical University Yachiyo Medical Center, Ohwada Shinden, Yachiyo City, Chiba, Japan; 2 Department of Biological Process of Aging, Tokyo Metropolitan Institute of Gerontology, Tokyo, Japan; 3 Research Institute of Vaccine Therapy for Tumours and Infectious Diseases, Nippon Medical school, Tokyo, Japan; 4 Division of Virology, National Cancer Center Research Institute, Tokyo, Japan; Albert Einstein College of Medicine, United States of America

## Abstract

More than 150 types of Human papillomaviruses (HPVs) have been isolated from numerous cutaneous and/or mucosal lesions. Flat wart samples on the face from 36 immunocompetent patients were collected and screened for HPV. From one sample, we cloned a putative novel genotype. The novel type consisted of 7779 bp in length with a GC content of 47.1%, containing open reading frames for putative early proteins (E1, E2, E4, E6, and E7) and two late proteins (L1 and L2). Homology searches and phylogenetic analyses indicated that it belonged to *Alphapapillomavirus* (*Alpha-PV*) species 2 and most closely resembled HPV 3. The virus fulfilled the definition of a novel type, and was named HPV 160 by the Reference Center for Papillomaviruses. The putative E7 protein of HPV 160 as well as HPV 29, 77, and 78 contained the Leu-X-Cys-X-Glu pRB-binding motif but other *Alpha-PV* species 2 (HPV 3, 10, 28, 94, 117, and 125) did not have this conserved motif.

## Introduction

More than 150 genotypes of Human papillomaviruses (HPVs) have been isolated from human cutaneous and mucosal lesions [1,2]. Novel HPV genotypes have less than 90% similarity of their L1 open reading frames (ORFs) with candidate known HPV genotypes [3,4]. The introduction of rolling circle amplification (RCA) for papillomaviruses [5] resulted in a significant increase in numbers of newly isolated and characterised HPV types. Based on the large number of partial sequences reported, many additional novel HPV genotypes after full sequence analyses are expected. To date, HPV genotypes belong to the five genera, *Alpha, Beta, Gamma, Mu*, and *Nu* according to the phylogenetic relationships of their complete L1 gene sequences [3,4]. HPV genomes with 60-70% similarities comprise different species, and less than 60% are considered different genera [4].

In the general population, cutaneous warts are caused by *Alpha-PV* species 2 (HPV 3, 10, 28, 29, 77, 78, 94, 117, and 125), species 4 (HPV 2, 27, and 57), *Mu*-PV (HPV 1 and 63), and *Nu*-PV (HPV 41). Among them, common warts are usually found on the hands and feet and caused by *Alpha-PV* species 4 [3,4], while flat warts, especially in young adults but sometimes in the elderly, are commonly found on the face, dorsal hands, or distal forearms and usually caused by *Alpha-PV* species 2 [3,4]. In the current study, flat warts on the face from 36 immunocompetent patients were screened for HPV, and a novel genotype HPV 160 belonging to *Alpha-PV* species 2 was isolated along with other genotypes belonging to *Alpha-PV* species 2 or 4.

## Materials and Methods

### Samples

Flat warts from 36 immunocompetent patients, presenting with brownish coloured skin on the face, were referred to Nippon Medical School and Tokyo Women’s Medical University Yachiyo Medical Center from 2008 to 2013. Diagnosis of flat warts was based on clinicopathological examination. The age of the patients ranged from 8 to 77 years, with a mean age of 36.1 years. Lesional biopsies were performed, and a part of the specimen was stored at -80°C for the HPV DNA test, and another part was used for histopathological examination. 

### Histopaholigical examination

The tissues were fixed with 10% formalin in PBS and then embedded in paraffin. Sections with a thickness of 3 μm were prepared by hematoxylin and eosin staining and subjected to microscopic examination. These studies were approved by the Ethics Committee of the Tokyo Women’s Medical University (Consent reference #2647), and written informed consent was obtained from each patient or his/her parents.

### PCR amplification

Two sets of consensus primers, SKF1/SKR1 and SKF2/SKR2 were used for PCR amplification to detect HPV genotypes from cutaneous warts as described previously [6]. PCR was performed in a total volume of 100 μL, containing 200-300 ng of cellular DNA from cutaneous warts, the above primers (40 pmol each), 2.5 units Ex Taq DNA polymerase (Takara, Tokyo, Japan), PCR buffer (50 mM KCl, 2 mM MgCl_2_, 10 mM Tris-HCl, pH 8.3), and 200 μM dNTPs. The PCR was performed under the following conditions: initial denaturation at 94°C for 4 min, 35 cycles with each cycle lasting 1 min, denaturation at 95°C, annealing at 50°C, extension at 72°C, and final elongation at 72°C for 4 min. Sequence analysis was performed on the amplified products, and HPV genotypes were identified by confirming more than 95% homology between the products and known HPV types. If the sequence homology showed lower than 90% to the closest related known HPV type, it was regarded as a putative new HPV type.

### Sequencing and cloning of the complete HPV 160 genome

Primers specific for HPV 160 were selected juxtaposing the NotI site located in the viral L1 gene. The primers were as follows: forward primer, 5′-aattGCGGCCGCGATGTCCCTGGTAGTGC-3′, and reverse primer, 5′- aattCGCGGCCGCTGTCACCCTTCATGTACAAGGCCTC-3′. The approximate 8 kb DNA segment was amplified by 30 cycles of PCR using KOD plus DNA polymerase (Toyobo, Tokyo, Japan) according to the manufacturer’s instructions. Amplified PCR products treated by NotI and NheI were cloned between NotI and SpeI sites of pBluescript II SK (-) (Stratagene, La Jolla, CA, USA). These cloned Notl-L and Nhel-S segments were sequenced by use of primers synthesized sequentially, according to the growing sequence information starting from both ends of the cloned HPV inserts as listed in Table S1. Sequencing reactions were performed on plasmid preparations of the two recombinant clones using the cycle sequencing reactions with BigDye Terminator v3.1 (Applied Biosystems, Carlsbad, CA, USA), and results were provided on an automated ABI model 3130xl sequencer following the manufacturer’s instructions. The nucleotide sequence was confirmed by sequencing two clones obtained from independent amplification reactions. The complete nucleotide sequence was submitted to the Reference Center PVs (Heidelberg, Germany), and then reconfirmed. Subsequently, the complete HPV 160 sequence became available in the DNA Data Bank of Japan (accession no. AB745694).

### ORF and phylogenetic analyses

ORFs of HPV 160 were predicted by using the ORF Finder (http://www.ncbi.nlm.nih.gov/projects/gorf/) and further confirmed by genome organization analysis by comparing with closely related HPV genotypes of L1 genes alignments using GENETYX software (version 11), and ClustalW program in BioEdit sequence alignment editor (version 7.1.11). Phylogenic relationships of HPV 160 with other *Alpha* ***-*** *PV*s were analysed and percentage similarities were calculated for *Alpha* ***-*** *PV*s species 2 of L1 ORF sequences by molecular evolutionary genetic analysis using the maximum likelihood method in the MEGA software (version 5.05) [7].

## Results and Discussion

**Figure 1:**
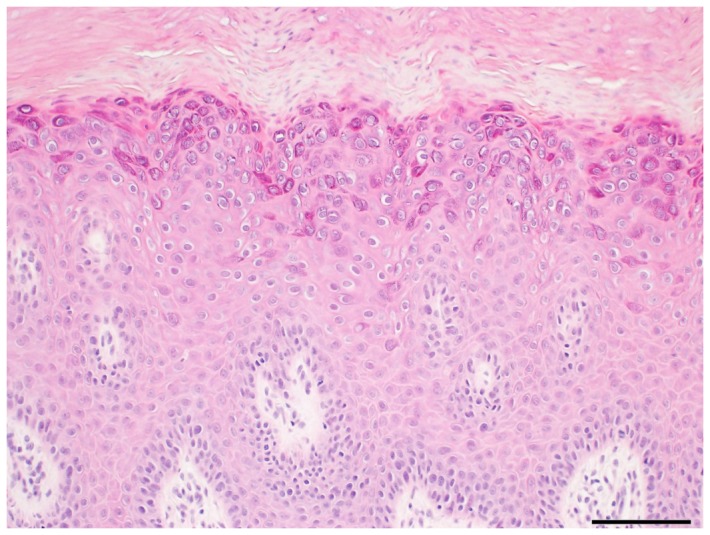
Histopathology of a flat wart from the face showing characteristic koilocytic changes in the upper epidermis with mild acanthosis. Hematoxylin and eosin staining, scale bar = 100 µm.

We examined 36 samples of flat wart tissues from the face, and tried to determine HPV genotypes causing the warts. SK-PCR designed to amplify *Alpha*-, *Gamma*-, and *Mu-*PVs with consensus primers [6] yielded DNA bands with estimated sizes in all samples. By sequencing, a single HPV type was identified in all of these samples, suggesting that sole HPV infection was common. The most common HPV type was HPV3 that was detected in 21 of 36 (58.3 %) samples. A secondary common type was HPV28 that was identified in four (11.1%) samples. HPV 94 and 29 were detected in three (8.3%) samples. HPV 2, 10, 27, 57 and a putative novel HPV genotype were each identified in one sample (Table 1). Among them, HPV 2, 27, and 57 belonged to *Alpha-PV*s species 4 while the others belonged to *Alpha-PV*s species 2, confirming that flat warts were not usually caused by *Alpha-PV*s species 2 [3,4], whereas HPV 77, 78, 117, and 125 were not found in the present study. Histological examination of the flat warts showed that the most characteristic cytopathic effect (CPE) was prominent koilocytic change in the granular cell layer (Figure 1). In our cases, three facial flat wart lesions containing HPV 2, 27, and 57 showed the presence of a small number of koilocytic changes without cytoplasmic inclusions in the upper epidermis; however, the differentiation of flat wart and common warts is sometimes histologically difficult. HPV DNAs were detected in two samples (HPV 3) without any significant CPEs, suggesting CPEs are not necessarily observed in flat wart lesions at least more than 10-years-old. 

**Table 1:** HPV Types Detected in Facial Flat Warts.

**HPV type**	**Lesion (*n*=36) no.**	**%**	**Genus**	**Species**
**HPV3**	**21**	**58.3**	***Alphapapillomavirus***	**2**
**HPV28**	**4**	**11.1**	***Alphapapillomavirus***	**2**
**HPV29**	**3**	**8.3**	***Alphapapillomavirus***	**2**
**HPV94**	**3**	**8.3**	***Alphapapillomavirus***	**2**
**HPV10**	**1**	**2.8**	***Alphapapillomavirus***	**2**
**HPV2**	**1**	**2.8**	***Alphapapillomavirus***	**4**
**HPV27**	**1**	**2.8**	***Alphapapillomavirus***	**4**
**HPV57**	**1**	**2.8**	***Alphapapillomavirus***	**4**
**HPV160**	**1**	**2.8**	***Alphapapillomavirus***	**2**

HPV, human papillomavirus

**Figure 2:**
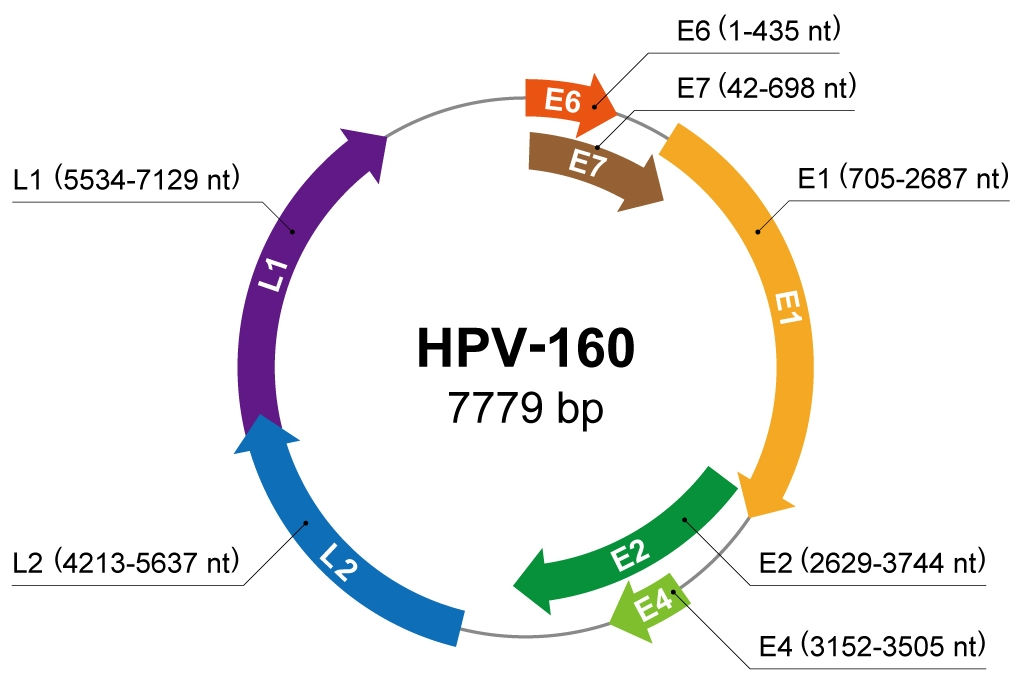
Genomic physical map of HPV 160 showing genomic positions of viral genes E6, E7, E1, E2, E4, L2, L1, and non-coding regions located between L1 and E6 (URR), and between E2 and L2.

**Figure 3:**
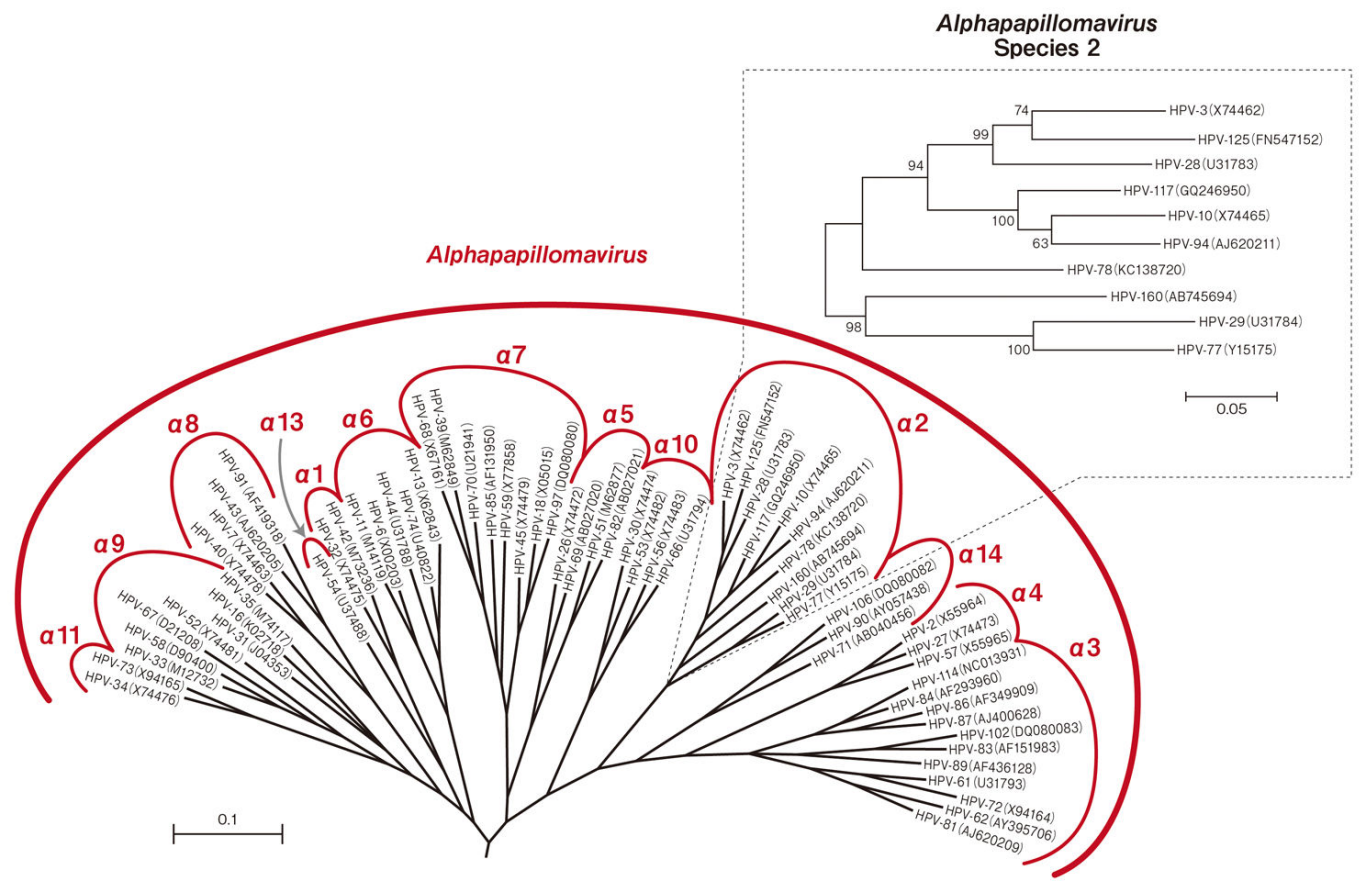
Phylogenetic tree based on L1 ORF sequences of HPV 160 and *Alpha-PVs*. The maximum likelihood method was used for analysis and the tree was constructed using MEGA software (version 5.05). The tree is drawn to scale, and the evolutionary distances were calculated in *Alpha-PVs*. Numbers show maximum likelihood bootstrap values above 50%.

The full genome of the putative novel HPV was cloned and sequenced as described in Materials and Methods, and the complete nucleotide sequence was deposited into the GenBank under the accession number AB745694 in 2012 (Figure 2). The Reference Center for PVs (Heidelberg, Germany) confirmed the complete sequence and numbered it as HPV 160. The complete genome of HPV 160 contained a total length of 7779 bp (GC ratio of 47.1%), conserved genome organization typical for HPVs, and contained ORFs for five early genes (E6, E7, E1, E2, and E4) and two late genes (L1 and L2) (Figure 2). Global alignment of the L1 sequence showed that this novel HPV genotype was most similar to HPV3, with 78.7% nucleotide homology and 86.0% amino acid sequence homology (Table S2). However, the nucleotide and amino acid sequences of L2, E6, and E7 were more similar to those of HPV29 and HPV77 than to HPV3 (Table S2). As shown in Figure 3, phylogenetic analysis using the maximum likelihood method based on L1 ORF sequences at the nucleotide level confirmed that HPV 160 belonged to the *Alpha-PV*s species 2. The non-coding upstream regulatory region (URR) was located between the L1 and E6 genes at nucleotide positions 7130 to 7779 (650 bp), and the other non-coding region was located between the E2 and L2 genes at nucleotide positions 3745 to 4212 (468 bp). The percentage similarities of nucleotide and amino acid sequences of six open reading frames of HPV 160 shared by other HPV genotypes belonging to *Alpha-PV*s species 2 are shown in Table S2.

E6 and E7 proteins of the high-risk HPVs bind and inactivate p53 and pRB, respectively [8-11]. Expression of both E6 and E7 is necessary for keratinocyte immortalisation and for maintaining proliferation in high risk HPV-associated tumours. The zinc-binding domain [CxxC(x)_29_CxxC] composed of two CxxC motifs separated from each other by 29 amino acids is conserved in E6 and E7 proteins of most PVs [12-14]. The zinc-binding domains of E6 and E7 proteins are thought to be involved in dimerisation and intracellular stabilization [15]. The putative E6 and E7 proteins of *Alpha-PV* species 2, including the novel HPV 160, conserve the typical zinc-binding domains (Figure S1a and b).

A conserved Leu-X-Cys-X-Glu (LxCxE) motif in HPV E7 was necessary and sufficient for E7 and pRB association [16-20]. E7 protein of not only high-risk HPVs but also large numbers of PVs binds pRB through a LxCxE motif [18,20] and inactivates it [17], suggesting that inactivation of pRB is probably essential for the life cycle of papillomaviruses. Among *Alpha-PV* species 2, HPV 160 as well as HPV 29, 77, and 78 conserve this pRB binding motif at alignment position 25-29, whereas HPV 3, 10, 28, 94, 117, and 125 do not (Figure S1a). In contrast, HPV 2, 27, and 57 have a conserved LxCxE motif in E7 proteins. As the lack of the LxCxE motif in E7 protein was generally rare among PVs [21], *Alpha-PV* species 2 is unique in consisting of these two subgroups. In the phylogenetic tree of *Alpha-PV* species 2, those which do not conserve this motif make one cluster, suggesting that the LxCxE motif was lost in the ancestral HPV of the cluster. It will be interesting to examine whether the E7 protein of this cluster (HPV 3, 10, 28, 94, 117, and 125) has completely lost the ability to bind and inactivate pRB. It will also be important to determine whether the E7 protein of the others (HPV 29, 77, 78, and 160) maintains significant activity to bind and inactivate pRB. Taking into account that the ability of E7 to inactivate pRB is associated with the proliferative capacity of the lesions [22] and that *Alpha-PV* species 2 caused non-proliferative flat warts lesions that may have regressed spontaneously, then inactivation of pRB may have become less essential for the life cycle of this species through evolution. In contrast, based on histological findings, flat warts appear to be similar, and prominent koilocytic changes without cytoplasmic inclusions are sometimes similar between *Alpha-PV* species 2 and 4 induced warts [2,23]. Therefore, it is difficult to distinguish the growth of the lesion based upon the presence of the LxCxE motif alone in flat warts.

## Conclusions

A novel HPV genotype HPV 160 was isolated from a flat wart lesion. Phylogenetic analysis indicated HPV 160 consisted of 7779 bp with a GC content of 47.1%, and belonged to *Alpha-PV* species 2. Among known HPVs belonging to *Alpha-PV* species 2, HPV 160 as well as HPV 29, 77, and 78 had a conserved LxCxE motif followed by a zinc-binding domain in the putative E7 protein. However, a difference in pRB binding activity of the E7 proteins among *Alpha-PV* species 2 remains to be elucidated.

## Supporting Information

Figure S1 **Amino acid alignment of HPV 160 E6 and E7 proteins with corresponding proteins of closely related genotypes from *Alpha-PVs* species 2 and genotypes HPV 6, 11, 16 and 18.** (**a**) The blue box indicates the location of the pRb-binding motif LxCxE of the E7 proteins conserved only in three genotypes (HPV 29, 77, and HPV 160) of *Alpha-PVs* species 2 as well as HPV 6, 11, 16 and 18. Red boxes indicate the C-terminal zinc-binding domain [CxxC(X)_29_CxxC] at alignment position 66-102. (**b**) Red boxes indicate two regular C-terminal zinc-binding domains [CxxC(X)_29_CxxC] at alignment positions 33-69, and 105-142 conserved in the E6 proteins.
(TIF)

Table S1 **Primers used for cloning and sequencing HPV-160.**
(DOC)

Table S2 **Primary sequence analysis of HPV 160 genes. Sequence percentage similarities between E6, E7, E1, E2, L1, and L2 genes of HPV 160 and closely related *Alpha-PVs* species 2.**
(DOC)
