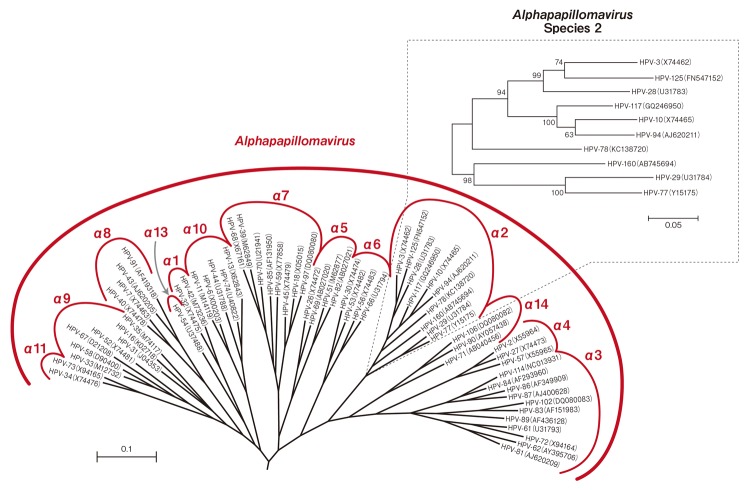# Correction: Molecular Cloning and Characterisation of a Novel Type of Human Papillomavirus 160 Isolated from a Flat Wart of an Immunocompetent Patient

**DOI:** 10.1371/annotation/effe849b-a0a8-4713-b1b0-96759ecafa65

**Published:** 2013-12-19

**Authors:** Tsuyoshi Mitsuishi, Ikuroh Ohsawa, Toshihiko Kato, Nagayasu Egawa, Tohru Kiyono

In Figure 3, alpha-6 and alpha-10 were erroneously switched. Alpha-6 should include HPV 30, 53, 56 and 66, and alpha-10 should include HPV 6, 11, 13, 44 and 74. Please see the corrected Figure 3 here: 

**Figure:**