# Supplementary figures and images for: Molecular Cloning and Characterisation of a Novel Type of Human Papillomavirus 160 Isolated from a Flat Wart of an Immunocompetent Patient
Source: PLoS One. 2013 Nov 8;8(11):e79592. doi: 10.1371/journal.pone.0079592 (PMC3835941; doi:10.1371/journal.pone.0079592)

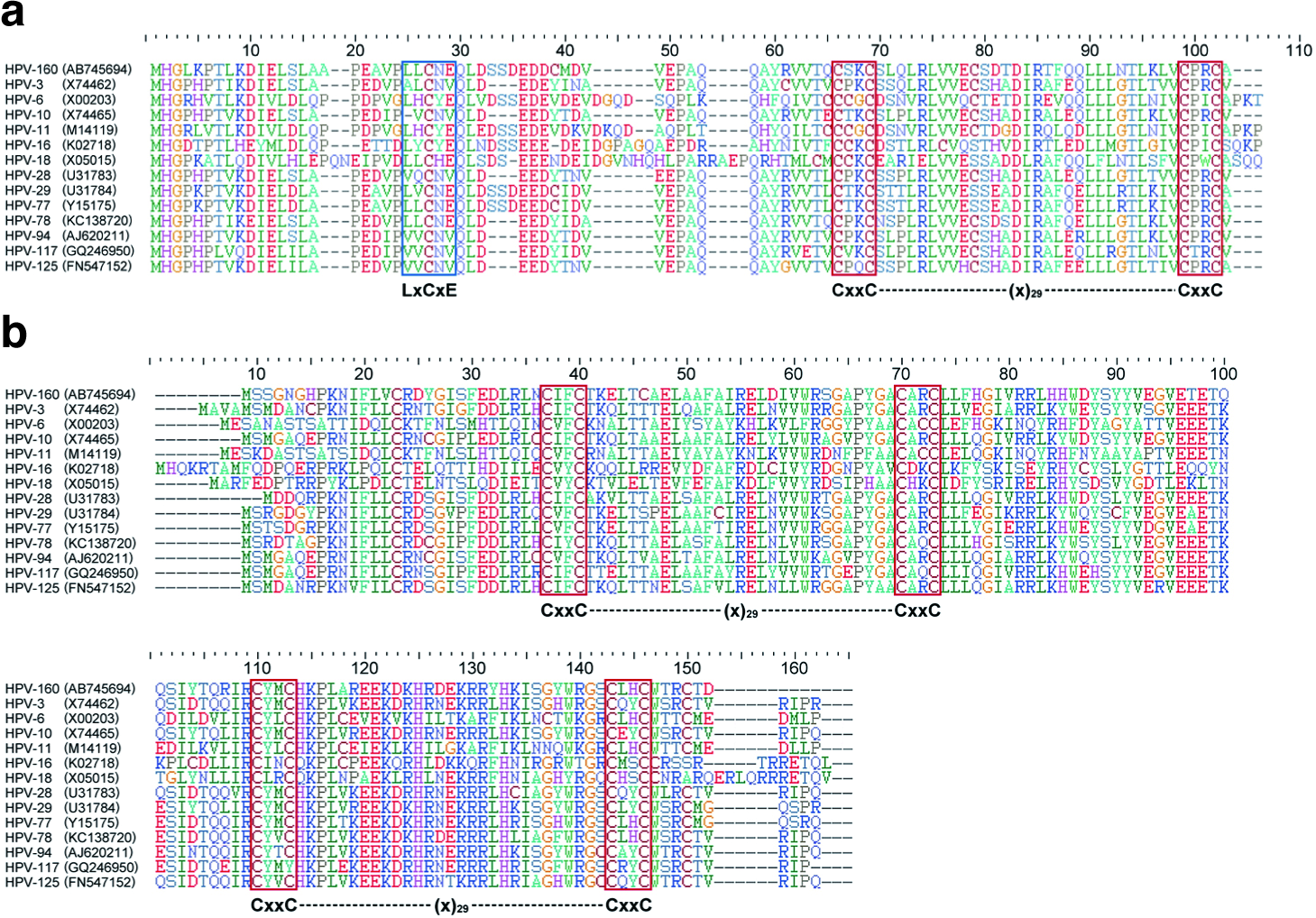

Supplement: Figure S1 — Amino acid alignment of HPV 160 E6 and E7 proteins with corresponding proteins of closely related genotypes from Alpha-PVs species 2 and genotypes HPV 6, 11, 16 and 18. (a) The blue box indicates the location of the pRb-binding motif LxCxE of the E7 proteins conserved only in three genotypes (HPV 29, 77, and HPV 160) of Alpha-PVs species 2 as well as HPV 6, 11, 16 and 18. Red boxes indicate the C-terminal zinc-binding domain [CxxC(X)29CxxC] at alignment position 66-102. (b) Red boxes indicate two regular C-terminal zinc-binding domains [CxxC(X)29CxxC] at alignment positions 33-69, and 105-142 conserved in the E6 proteins. (TIF) [file pone.0079592.s001.tif]
