# Supplementary material for: Molecular Cloning and Characterisation of a Novel Type of Human Papillomavirus 160 Isolated from a Flat Wart of an Immunocompetent Patient
Source: PLoS One. 2013 Nov 8;8(11):e79592. doi: 10.1371/journal.pone.0079592 (PMC3835941; doi:10.1371/journal.pone.0079592)
Supplement: Table S1 — Primers used for cloning and sequencing HPV-160. (DOC) [file pone.0079592.s002.doc]

| **Table S1. Primers used for sequencing HPV-160** | | | |
| --- | --- | --- | --- |
|  | Sequence (5' →3') | Position | Clone |
| SKF1 | AAATATCCAGATTATCTRAARATG | 6408-6431 | Ref 6 |
| SKF2 | AAATATCCTGATTATTTRGGMATG | 6408-6431 | Ref 6 |
| SKR1 | ATACCATAGAYCCACTRGG | 6621-6603 | Ref 6 |
| SKR2 | AAACYATAGAGCCACTWGG | 6621-6603 | Ref 6 |
|  | Sequence (5' →3') | Position | Clone |
|  |  |  | NheI-S |
| F2 | GGTACCGTTCTCGATCTG | 7035-7052 |  |
| F3 | ATCCGGTGCGACCGAATG | 7721-7738 |  |
| F4 | GGTTGTAGAGTGCAGCGAC | 611-629 |  |
|  |  |  |  |
| R9 | GCCCGTTCCGCCTCAGTGC | 757-739 |  |
| R10 | GGTCCTCAAATGATATGCC | 73-55 |  |
| R11 | GTACAAAACAGGAACTAAAC | 7163-7144 |  |
|  |  |  | NotI-L |
| F6 | TAGGGATGCTTGCACAATG | 1943-1961 |  |
| F7 | TGAAACAGATAGCAACAAAC | 2684-2704 |  |
| F8 | CTATGTGTATGGGGAGACT | 3159-3177 |  |
| F9 | CGCCCTGGATTTTTACCTGT | 3765-3784 |  |
| F10 | GATGTCAGTGTTCCTGCTC | 4459-4477 |  |
| F11 | GATTCAGTCGTCTGGGTCAG | 5108-5127 |  |
| F12 | TAAGGTGTCTGCTTTTCAG | 5809-5827 |  |
|  |  |  |  |
| R6 | ACATAGGCATCATCTGCA | 3164-3147 |  |
| R5 | TAGCACGTAAGCGCAGGA | 3863-3846 |  |
| R4 | GCTAGAGTCCTCCAACAAG | 4548-4530 |  |
| R3 | CAGGTATAGGGCTAAGATC | 5206-5188 |  |
| R2 | GGTTATAGATGCGTGCATC | 5897-5879 |  |
| R7 | CTGTGCTGTGAATGGTAGGG | 2528-2509 |  |
| R8 | AACCGTATCCATTCAGACAT | 2020-2001 |  |
| R2-2 | GGAAAGGGCGGAAGGAAAC | 5388-5370 |  |
| F; forward primer; R, reverse primer | |  |  |
