# Supplementary material for: Molecular Cloning and Characterisation of a Novel Type of Human Papillomavirus 160 Isolated from a Flat Wart of an Immunocompetent Patient
Source: PLoS One. 2013 Nov 8;8(11):e79592. doi: 10.1371/journal.pone.0079592 (PMC3835941; doi:10.1371/journal.pone.0079592)
Supplement: Table S2 — Primary sequence analysis of HPV 160 genes. Sequence percentage similarities between E6, E7, E1, E2, L1, and L2 genes of HPV 160 and closely related Alpha-PVs species 2. (DOC) [file pone.0079592.s003.doc]

| **Table S2. Primary sequence analysis of HPV-160 genes. Sequence percentage similarities between E6, E7, E1, E2, L1, and L2 genes of HPV-160 and closely related species 2 of *alpha papillomaviruses* species 2** | | | | | | | | | |
| --- | --- | --- | --- | --- | --- | --- | --- | --- | --- |
|
|
| **HPV160** | **HPV-3** | **HPV-10** | **HPV-28** | **HPV-29** | **HPV-77** | **HPV-78** | **HPV-94** | **HPV-117** | **HPV-125** |
| **E6** |  |  |  |  |  |  |  |  |  |
| nt | 74.4 | 76.7 | 76.7 | 75.5 | 77.3 | 76.7 | 76.0 | 74.0 | 73.3 |
| aa | 72.7 | 76.2 | 74.3 | 78.2 | 76.8 | 72.7 | 73.4 | 72.0 | 68.5 |
| **E7** |  |  |  |  |  |  |  |  |  |
| nt | 77.5 | 80.8 | 79.3 | 85.1 | 85.5 | 83.3 | 80.8 | 78.3 | 77.2 |
| aa | 71.1 | 72.2 | 68.9 | 80.2 | 81.3 | 76.7 | 73.3 | 65.6 | 65.9 |
| **E1** |  |  |  |  |  |  |  |  |  |
| nt | 81.1 | 72.3 | 81.4 | 80.8 | 80.0 | 81.1 | 72.7 | 71.9 | 80.9 |
| aa | 82.3 | 80.2 | 83.4 | 84.2 | 82.7 | 84.0 | 82.1 | 79.9 | 80.8 |
| **E2** |  |  |  |  |  |  |  |  |  |
| nt | 75.7 | 77.5 | 77.8 | 69.0 | 68.5 | 78.5 | 77.9 | 75.7 | 76.8 |
| aa | 68.6 | 73.4 | 73.1 | 70.4 | 71.8 | 74.3 | 74.2 | 70.2 | 70.6 |
| **L1** |  |  |  |  |  |  |  |  |  |
| nt | 78.7 | 76.5 | 77.6 | 76.6 | 77.2 | 78.6 | 78.1 | 78.3 | 76.9 |
| aa | 86.0 | 83.6 | 83.3 | 83.6 | 82.9 | 86.0 | 82.6 | 84.8 | 83.8 |
| **L2** |  |  |  |  |  |  |  |  |  |
| nt | 74.8 | 75.5 | 75.8 | 76.2 | 76.8 | 75.6 | 73.8 | 74.7 | 74.8 |
| aa | 79.5 | 78.5 | 78.7 | 80.6 | 81.4 | 78.9 | 77.4 | 78.4 | 79.0 |
| **nt; nucleotide, aa; aminoacid** | | |  |  |  |  |  |  |  |
